# Toxic encephalopathy and peripheral neuropathy of poisoning by Avermectin Pyridine: a case report and a review of the literature

**DOI:** 10.3389/fneur.2023.1144970

**Published:** 2023-06-02

**Authors:** Jiuzhou Diao, Qingbo Zhou

**Affiliations:** Department of Neurology, The Second Hospital of Shandong University, Jinan, China

**Keywords:** Avermectin and Pyridine, toxic encephalopathy, peripheral neuropathy, therapy, case report

## Abstract

**Background:**

Avermectin Pyridaben (AVP) is an insecticide with extreme neurotoxicity in human, causing critical symptoms such as nausea, vomiting, coma and respiratory failure within a short time after oral ingestion. Neurological sequelae or even death may occur because of delayed treatment or excessive toxic dose.

**Case presentation:**

We report a 15-year-old girl who presented with coma, respiratory failure, limb weakness, ataxia symptoms after ingestion of a toxic dose of AVP. Soon after the poisoning, the patient was treated with life-saving mechanical ventilation and haemodialysis. Subsequently brain Magnetic Resonance Imaging (MRI) and nerve conduction study (NCS) and electromyography (EMG) demonstrated toxic encephalopathy and peripheral nerve injury. Over the next 2 months the patient’s limb function gradually recovered under treatment with hyperbaric oxygen, glucocorticoid pulses and neurotrophic drugs.

**Conclusion:**

This case documents a rare presentation of toxic encephalopathy complicated with peripheral neuropathy following AVP poisoning. Seven other similar cases of poisoning in terms of common symptoms and effective treatment have also been summarised for providing clinicians with experience in diagnosis and therapy.

## Introduction

As a pesticide commonly used in agriculture, Avermectin Pyridaben is a mixed preparation consisting of 0.3% Avermectin and 10.2% Pyridaben. Avermectin which can be extracted from fermenting of *Streptomyces avermitilis*, presents the chemical structure of a large ring lactone compound, killing insects by the effect of preventing the transmission of electrical impulse which stimulate gamma-aminobutyric acid (GABA) receptors and paralysing nerves (1). Pyridaben is a highly lipohpilic compound with higher bioactivity, lower toxicity, and excellent selectivity compared to benzenoid (2) and can be generated by Intermediate Derivatization Methods (IDM) and terminal group replacement method (TRM) (3), it also can induce neurotoxicity by inhibiting mitochondrial respiration and disrupting oxidative stress and ubiquitin-proteasome system dysfunction (4, 5). The Food and Agriculture Organization of the United Nations (FAO) states that the human intake of Pyridaben should not exceed 0.405 mg/kg in 1 day. With the widespread use of AVP in agriculture, there has been a gradual increase in the incidence of pesticide poisoning, which in severe cases can be life-threatening. This paper reports a case of toxic encephalopathy with peripheral nerve injury after AVP poisoning, which recovered after treatment with hyperbaric oxygen, hormone and neurotrophic therapy. In addition, the symptoms and treatment of seven other similar poisoning cases are discussed in this paper, aiming to increase awareness of the safety of using AVP and to summarise experience in the diagnostic treatment of this type of poisoning.

## Case report

A female adolescent aged 15 years manifested symptoms of nausea, vomiting, dyspnea and unconsciousness an hour subsequent to the inadvertent ingestion of 50 mL of AVP (comprising 0.3% Avermectin and 10.2% Pyridaben) at home. Two hours later she experienced a state of shock and was admitted to a nearby hospital. She had no previous medical history. Her vital signs showed dilated, non-reactive pupils and no spontaneous breathing. Her blood pressure (BP), pulse rate (PR), respiratory rate (RR), temperature and blood glucose (BS) were 65/30 mmHg, 74 bpm, 18/min with balloon-assisted ventilation, below 35°C, and 26.4 mmol/L, respectively. An arterial blood gas (ABG) analysis showed metabolic acidosis with respiratory alkalosis (pH = 6.77, pO_2_ = 242 mmHg, pCO_2_ = 26.2 mmHg, HCO_3_ = 12.3 mmol/L, BE = -15 mmol/L, Lactate beyond the max range). The 12-lead electrocardiogram was normal. Other laboratory exams are presented in Table 1.

**Table 1:** On-arrival laboratory test results.

Laboratory test	Result (normal range)
White blood cells (×1,000/mm^3^)	11.2 (4.5–11)
Hemoglobin (g/L)	97 (110–150)
Hematocrit (percent)	32 (35–45)
Platelet count (×1,000/mm^3^)	448 (150–450)
Urea (mmol/L)	4.0 (1.7–8.3)
Creatinine (umol/L)	39 (40–84)
Aspartate transaminase (U/L)	18 (0–40)
Alanine transaminase (U/L)	20 (6–29)
Lactate dehydrogenase (U/L)	229 (80–240)
Creatine phosphokinase (U/L)	43 (0–180)
Alkaline phosphatase (IU/L)	80 (15–140)
Total bilirubin (umol/L)	6.8 (5.1–28)
Direct bilirubin (umol/L)	1.1 (0–10)
Serum sodium (mmol/L)	136 (131–148)
Serum potassium (mmol/L)	3.9 (3.5–5.3)
Prothrombin time (seconds)	11.3 (10–14)
International normalized ratio	0.94 (0.8–1.5)
Partial thromboplastin time (seconds)	30 (22–38)
Reticulocytes	1.4 (0.5–1.5)
Arterial PH	6.77 (7.35–7.45)
Arterial pCO2 (mmHg)	26.2 (35–45)
Arterial pO2 (mmHg)	242.5 (90–100)
Arterial HCO3 (mmol/L)	12.3 (22–27)

Haemodialysis and mechanical ventilation were adopted in the treatment and, in the meantime, norepinephrine (0.2 μg/kg/min) was used to maintain the blood pressure. Having been transferred to ICU, she was treated with normal saline (1 L/d), intravenous pantoprazole (0.1 g q12h) and citicoline (0.25 g qd) which could protect gastic mucosa and the brain. On next day of admission, she regained consciousness, but still be weak, felt powerless and be in mechanical ventilation requirement. Her neurological examination revealed positive Babinski signs, grade IV muscle strength and ataxia in both lower limbs, and no ataxia, grade V muscle strength in both upper limbs, and no abnormalities on sensory examination such as depth sensation, positional sensation, pain and temperature sensation in the limbs. The examination suggested her pyramidal tract involvement, which prompted a request for a brain MRI, but it was not done due to her unpredictable condition. On the day 4 of ICU treatment, the patient was extubated and transferred to the general ward for continued treatment on the day 5. A list of vital signs care during ICU treatment can be found in Table 2. After ten-day treatment at the local hospital, the patient’s BP, PR, RR, BS, O2 saturation, temperature, and ABG indicators returned to the normal range. Then she was transferred to our hospital for lower limb ataxia and positive Babinski signs, which suspected neurological impairment and confirmed by brain MRI NCS and EMG.

**Table 2:** Changes in vital signs in the intensive care unit within 5 days after poisoning.

	1	2	3	4	5
BP(mmHg)	65/30	98/67	101/65	104/68	96/66
R(times/min)	18	18	18	18	17
T(°C)	35	36.1	36.3	36.5	36.4
P(bpm)	74	70	68	69	67
Life support interventions	MV + VA	MV + VA	MV	MV	/

BP, blood pressure; R, respiratory frequency per minute; T, body temperature; P, pluse rate; MV, mechanical ventilation; VA, vasoactive agent.

**Figure 1:**
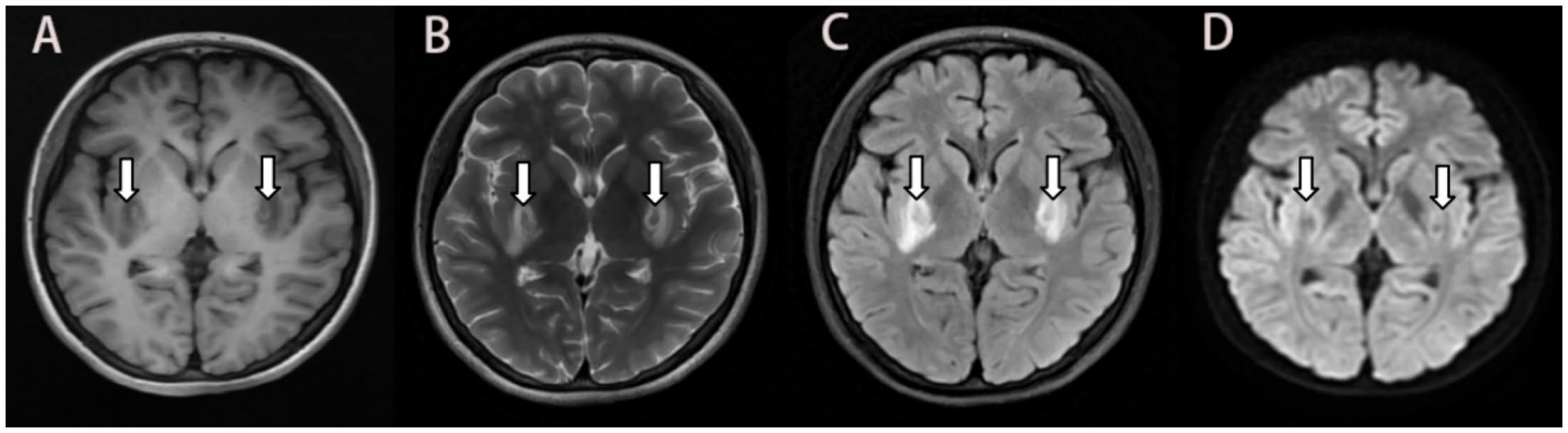
MRI 10 days after ingestion showing bilateral symmetrical lesions in the basal ganglia. Abnormal isointensity on T1-FLAIR **(A)**, and heterogeneous intensity on T2 propeller **(B)**, and hyperintensity on T2-FLAIR **(C)**, and no significant hypersignal on DWI **(D)**. MRI: magnetic resonance imaging; FLAIR: fluid attenuated inversion recovery; DWI: diffusion-weighted imaging.

Her brain MRI showed abnormal signals in the internal and external capsules of the basal ganglia bilaterally, confirming toxic encephalopathy (Figure 1). Combined with the patient’s respiratory failure after poisoning, the possibility of hypoxia-induced encephalopathy cannot be ruled out. NCS and EMG evoked potential testing suggested peripheral neuropathy of both lower limbs, including axonal damage to motor and sensory nerves (Table 3). Based on her weight of 52 kg the management of her neurological impairment was as follows: Intravenous dexamethasone sodium phosphate injection was given for 11 days (10 mg*5 days, 7.5 mg*3 days, 5 mg*3 days), then switched to oral methylprednisolone tablets with an initial dose of 40 mg/d, and the dose was reduced to one slice a week until the drug was stopped. Glucocorticoid therapy was administered for a duration of 3 months in order to mitigate the effects of inflammation and cerebral oedema. Hyperbaric oxygen (HBO) therapy lasted for 10 days with parameters set at 0.25 MPa and 60 min/d to reduce cerebral hypoxia, but was eventually discontinued due to financial constraints (6). Mecobalamin (0.5 gr qd) and vitamin B1 (0.1 gr qd) injections were used to promote regeneration of peripheral injured nerves and continued for 1 month (7, 8). The patient complied with medical advice and did not have any adverse effects during treatment.

**Table 3:** The amplitude (Amp) of sensory and motor neuropathy in NCS and EMG.

Amp	Suralis sensory (μV)			Peroneus Motor (mV)		
Stimulation times	T1	T2	T3	T1	T2	T3
RT	6.4↓	3.1↓	2.8↓	5.0	8.1	4.7
LT	5.9↓	3.8↓	2.2↓	0.4↓	0.9↓	0.5↓

RT, right lower limb; LT, left lower limb; T1, 10 days after poisoning; T2, 2 months after poisoning; T3, 4 months after poisoning. The sensory nerve stimulation site was the ankle and the recording site was the foreleg. The motor nerve stimulation site was fibula superior and the recording site was extensor digitalis brevis. Conclusion: NCS and EMG-T1 showed axonal damage in the sensory nerves of the both lower limbs and the motor nerves of the left lower limb. NCS and EMG-T2 and T3 showed axonal damage in the sensory nerves of the both lower limbs. Due to the different machines used in each NCS and EMG test, the accuracy of stimulation site, depth and recording time will vary between operators. The measured NCS and EMG value can only be used as a qualitative criterion for the presence or absence of nerve damage and cannot used as a quantitative assessment index of improvement or deterioration. Therefore, the results of the NCS and EMG report should be taken as the final standard.

**Figure 2:**
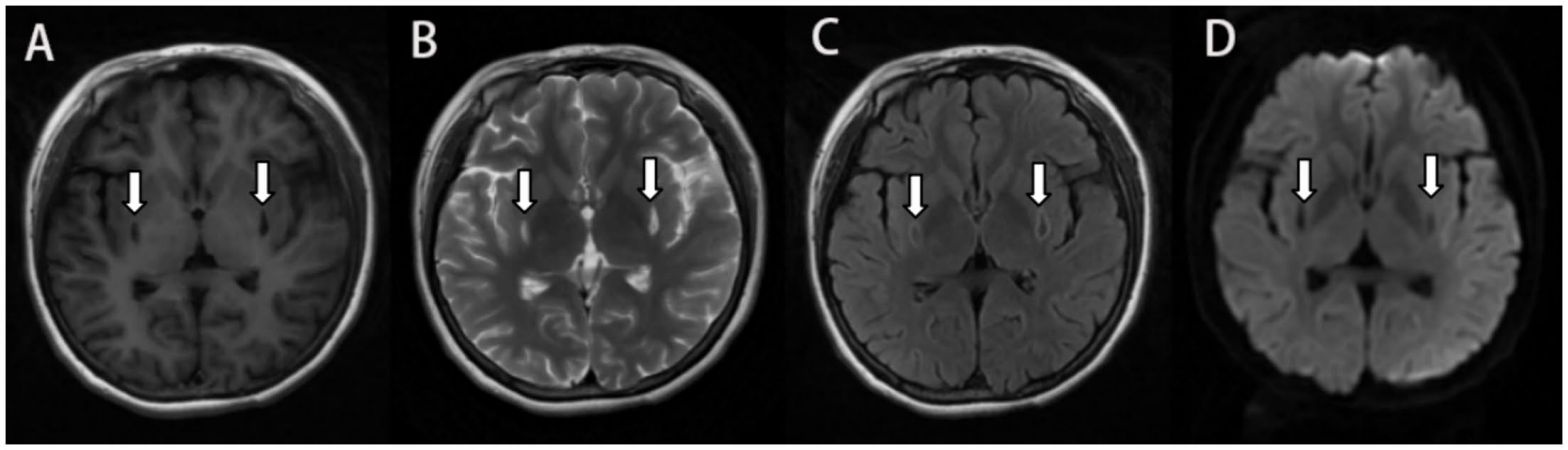
MRI 2 months after ingestion showing encephalomalacia in the bilateral basal ganglia with surrounding gliosis and the lesion size was smaller than before. Symmetrical small patchy with long T1 long T2 signal on T1 Flair **(A)** and T2 propeller **(B)**, and hyperintense in the edge of the lesion on T2-FLAIR **(C)**, and no significant hypersignal on DWI **(D)**.

**Figure 3:**
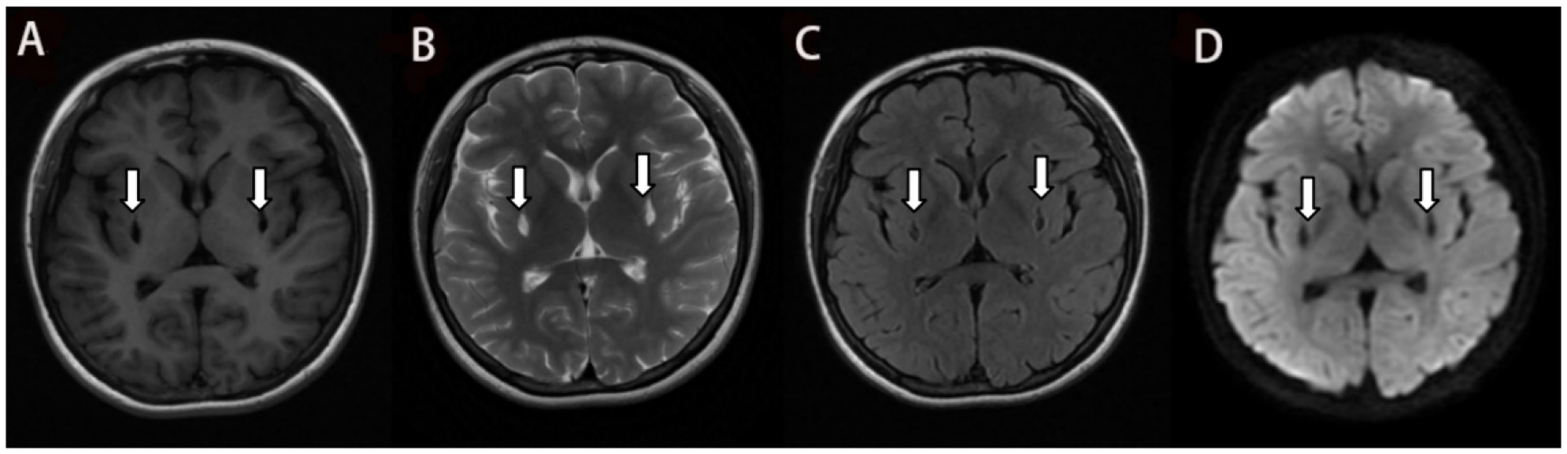
The T1 Flair **(A)**, T2 Propeller **(B)** and DWI **(D)** in the MRI 4 months after ingestion show similar to Figure 2. and the gliosis around the lesion is less than before which is shown in the T2 Flair **(C)**.

She was reexamined by brain MRI, NCS, and EMG 45 days after glucocorticoid treatment and 4 months after poisoning, respectively. The brain MRI showed a remarkable reduction in the size of the lesions and the FLAIR hyperintensities in both basal ganglia regions, together with the observation of gliosis in the adjacent affected areas (Figures 2, 3), but NCS and EMG showed persistence of motor and sensory axonal damage in the lower limbs (Table 3). Her neurological examination showed a positive Babinski sign, no ataxia and normal muscle strength of the limbs.

## Discussion

Avermectin can stimulate gamma-aminobutyric acid (GABA) receptors in the central nervous system to produce inhibitory neurotransmitters (9) and reduce cell viability and adenosine triphosphate (ATP) production by affecting mitochondrial function, thereby impairing the function of other organelles, leading to an imbalance in intracellular calcium homeostasis and eventually leading to cell necrosis (10). Pyridaben, an inhibitor of mitochondrial oxidation complex I, can significantly increase the production of reactive oxygen species (ROS) during part of the action period (11), which can induce apoptosis by affecting mitochondrial function, inducing ROS, inducing calcium imbalance, increasing the endoplasmic reticulum stress response, and promoting apoptosis (12). The patient reported taking 50 mL of a mixture of Avermitin 150 mg and Pyridine 15,100 mg and was found to have symptoms of toxic encephalopathy, and Peripheral neuropathy was found the next day after rescue. This is similar to the case of Avermectin poisoning with neurological injury-related sequelae reported by Song, which suggests that simultaneous central and peripheral neuropathy may have a poor prognosis (13). The poison reaches the brain tissue through the blood, inhibits mitochondrial function, and leads to tissue hypoxia and necrosis. Gray matter is more vulnerable to anoxic or ischemic insults due to its higher metabolic demands for oxygen and glucose, and deep gray nuclei are often involved in acute severe brain tissue hypoxia, which is associated with poor prognosis (14–16).

This paper presents a discussion regarding a total of eight patients who suffered from poisoning, each exhibiting varying degrees of symptoms related to neurological damage include ataxia, muscle weakness, coma, blepharoptosis, hypoesthesia and myoclonus et al. (Table 4) (13, 17–22). In addition to nervous system symptoms, gastrointestinal irritation symptoms were common, and more than half of the patients had respiratory system involvement. One patient had allergic symptoms, and another one had cerebral haemorrhage, multiple organ failure and eventually died. The dose of poisoning, the rescue time and the presence or absence of major organ damage were the main factors influencing prognosis.This paper presents an overview of treatment methods for AVP poisoning, as no singular antidote for its mitigation is currently available.

**Table 4:** Summary of 8 cases of AVP related poisoning.

	No. 1	No. 2	No. 3	No. 4	No. 5	No. 6	No. 7	No. 8
Author	Karatelike H	Wenjie W	Aminiahidashti H	Sung YF	Soyuncu S	Jiezheng D	Jianian D	Our case
Age/Sex	30/M	47/M	42/M	76/M	25/W	28/W	28/W	15/W
Pesticide-dose	A-12 mg/kg	A u	A-51.42 mg/kg	A-414.2 mg/kg	A-108 mg/kg	A + P-100 mL	P-20 mL(15%)	0.3%A + 10.2%P-50 mL
Onset/Escape	<3 h/27 h	N/5 h	2.5 h/24 h	1 h/9 days	<3 h/u	2 h/16 h	2 h/Death	1 h/24 h
GAS	9	10	u	3	6	4	u	3
BP-PO2(mmHg)	120/60-u	135/90-u	80/65-u	94/49-195(MV)	102/59–68.5	85/54–58	65/26–33	65/30–242.5(MV)
Neurological	Ataxic, Blepharoptosis	/	Muscle weakness, Myoclonus	Coma, Myoclonus, Muscle weakness, Peripheral neuropathy	/	Coma	Coma, Cerebral hemorrhage, Toxic encephalopathy	Coma, Ataxic, Muscle weakness, Peripheral neuropathy, Toxic encephalopathy
Other symptoms	u	u	Fever, Diarrhea, Allergy	RF	N/V, RF	N/V, Pulmonary infection	N/V, MOF	N/V, RF, Shock
Main treatments	GL	GL, MV	GL, VA, H1-R blocker, H2-R blocker, Hormone	GL, VA, MV, H2-R blocker, Hormone	GL, MV	GL, MV, Anticholinergic, Antibiotics, Neurotrophic	GL, VA, Hemodialysis, CRRT	Enema, MV, VA, Hemodialysis, Hormone, Neurotrophic
Prognosis	R	R	R	S	R	R	D	R

M, Man; W, Woman; A, Avermectin; P, Pyridaben; u, unreported; H, Hypoactive; N, Negative; Onset/Escape, the time of onset and escape from life risk; N/V, Nausea/Vomiting; RF, Respiratory failure; MOF, Multiple organ function; GL, Gastric lavage; VA, vasoactive agent; MV, mechanical ventilation; CRRT, Continuous renal replacement therapy; R, Clinical Recovery; S, Severe sequelae; D, Death.

Toxins should be removed from the body as soon as possible after poisoning. Activated charcoal gastric lavage, intestinal perfusion, hemodialysis and other methods could be used to avoid the occurrence of MOF. At the same time, vasoactive drugs were used to maintain blood pressure in the normal range, and mechanical ventilation support was given to patients with dyspnea. Patients with toxic encephalopathy can use glucocorticoid to control the inflammatory response of cells, reduce brain edema, and prevent compression of the surrounding normal brain area. Hyperbaric oxygen therapy (HBO) inhibits neutrophil adherence to the walls of ischemic vessels, which decreases free radical production, vasoconstriction and tissue destruction (14). Patients with Peripheral neuropathy should be given timely nutritional nerve treatment to prevent irreversible sequelae. Liver damage, pulmonary infection, gastrointestinal ulcer, and allergy are all possible complications, and doctors need to predict the possible situation to reduce the incidence of complications.

## Data availability statement

The original contributions presented in the study are included in the article/supplementary material, further inquiries can be directed to the corresponding author.

## Ethics statement

The studies involving human participants were reviewed and approved by Research ethics committee of the second hospital of shandong university. Written informed consent was obtained from the individual(s), and minor(s)' legal guardian/next of kin, for the publication of any potentially identifiable images or data included in this article.

## Author contributions

JD collected the clinical information, and examination data and wrote the manuscript. QZ provides thesis guidance and financial support. All authors contributed to the article and approved the submitted version.

## Funding

Supported by Natural Science Foundation of Shandong Province of China (ZR2021MH098).

## Conflict of interest

The authors declare that the research was conducted in the absence of any commercial or financial relationships that could be construed as a potential conflict of interest.

## Publisher’s note

All claims expressed in this article are solely those of the authors and do not necessarily represent those of their affiliated organizations, or those of the publisher, the editors and the reviewers. Any product that may be evaluated in this article, or claim that may be made by its manufacturer, is not guaranteed or endorsed by the publisher.
